# Editorial: Multi-modal imaging and natural products nanomedicine platform for advancing imaging-guided diagnosis and therapeutics

**DOI:** 10.3389/fbioe.2026.1804653

**Published:** 2026-05-01

**Authors:** Shiying Li, Wenlin Chen, Qian Huang

**Affiliations:** 1 Hong Kong Polytechnic University, Kowloon, Hong Kong SAR, China; 2 Great Bay University, Dong Guan, China; 3 Wuhan Textile University, Wuhan, China

**Keywords:** advancing imaging-guided, diagnosis and therapeutics, multi-modal imaging analysis, nanoparticle, natural products

## Introduction

The landscape of modern precision medicine is increasingly defined by the synergy between high-sensitivity diagnostic imaging and targeted therapeutic intervention. By integrating the structural and functional insights of multi-modal imaging with the biocompatibility and pharmacological potency of natural products, bioengineers are now constructing “theranostic” platforms of unprecedented sophistication. This Research Topic highlights five strategic contributions that exemplify this trajectory—bridging the gap between mutation-specific PET imaging, biomimetic drug delivery, and microenvironment-responsive scaffolds.

### Precision diagnostics: the strategic baseline

Effective imaging-guided therapy necessitates a deep understanding of the molecular evolution of disease. A recurring challenge in oncology is the dynamic emergence of drug resistance, such as the EGFR T790M mutation in non-small-cell lung cancer (NSCLC). Xie et al. address this by reporting a novel ^68^Ga-labeled tyrosine kinase inhibitor (TKI) probe.

By shifting from static, invasive biopsies to real-time, non-invasive PET imaging, this work provides a scalable methodology to monitor tumor evolution and synchronize therapeutic pivots with the tumor’s current genetic profile.

This diagnostic precision is further expanded in the realm of interventional oncology, where the localization of treatment is as critical as the diagnosis itself. Jiang et al. offer a critical evaluation of radioactive microspheres for brachytherapy. Their synthesis transcends basic technical reporting, identifying key requirements for the next-generation of multifunctional microspheres—specifically, the need for enhanced radionuclide containment coupled with high-fidelity imaging visibility. This “see-and-treat” capability ensures dosimetric accuracy in solid tumors, allowing for personalized dose adjustments based on real-time imaging feedback, thereby minimizing damage to adjacent healthy tissues.

### Biomimicry and the natural Product reservoir

Natural products remain an indispensable source of therapeutic diversity, yet their clinical utility is frequently limited by unfavorable pharmacokinetics, poor solubility, and rapid systemic clearance. Zhuang et al. demonstrate how biomimetic engineering can circumvent these barriers by utilizing K562 cell membranes to encapsulate vinca alkaloids derived from *Catharanthus roseus*. These “cloaked” vesicles leverage the natural interface of the cell membrane to evade immune clearance and improve tumor accumulation. By transforming potent natural cytotoxic agents into targeted nanomedicines, this research provides a template for reducing the systemic footprints of highly active natural compounds while maximizing their concentration at the site of malignancy.

Beyond oncology, this platform approach is proving transformative for cardiovascular health, illustrating the versatility of natural product-based nanomedicine. Vascular calcification, once considered an intractable degenerative process, is now being targeted through specific pharmacological pathways identified through advanced screening. Mo et al. identify isowighteone, a flavonoid from *Ficus hispida*, as a promising inhibitor of vascular mineralization. Their mechanistic study elucidates how targeting the HSP90AA1-mediated PI3K-Akt pathway can suppress osteogenic gene expression in vascular smooth muscle cells. This work provides a molecular blueprint for using natural products not just as supplements, but as precision tools in cardiovascular care.

### Microenvironment-responsive architectures

The success of any imaging-guided platform ultimately depends on the responsiveness of the carrier material to the biological context. A static carrier often fails to release its payload effectively or releases it prematurely, leading to sub-optimal therapeutic outcomes. Li and Li present a composite hydrogel system that integrates GSH-responsive nanoparticles into a silk fibroin matrix.

This architecture is designed to remain stable under physiological conditions until it encounters the specific redox microenvironment characterized by high glutathione (GSH) levels in diseased tissue. This encounter triggers a localized, burst-free release of the therapeutic payload. Such stimuli-responsive scaffolds represent a critical step toward integrated platforms that serve as both structural supports for tissue regeneration and intelligent reservoirs for drug delivery, bridging the gap between regenerative medicine and precision pharmacology.

### The synergy of multi-modal platforms

The articles collected in this Research Topic underscore a fundamental shift in bioengineering: the move away from isolated diagnostic or therapeutic agents toward integrated, multi-modal platforms. The convergence of PET/CT imaging agents with biomimetic carriers ensures that we are not only treating the right target but doing so at the right time in the disease’s evolutionary cycle. By utilizing natural products, these platforms also benefit from reduced immunogenicity and novel mechanisms of action that are often superior to purely synthetic alternatives.

Furthermore, the integration of imaging-guided navigation allows for a level of surgical and interventional precision previously unattainable. Whether it is verifying the accumulation of cell-membrane vesicles in a tumor or monitoring the degradation of a responsive hydrogel, the “imaging-guided” aspect of these platforms provides a crucial layer of safety and verification. This is particularly relevant as we move toward more complex therapies, such as internal irradiation and gene-targeted pharmacology, where the margin for error is narrow.

## Conclusion

The collective research presented here highlights that the future of advancing diagnosis and therapeutics lies in the convergence of multi-modal imaging and innovative nanomedicine platforms. From mutation-specific tracers to cell-membrane-shrouded natural products and smart hydrogels, these studies provide the foundational tools for a more agile and personalized healthcare system. We hope this collection serves as a significant reference for researchers working at the intersection of bioengineering, molecular imaging, and natural product pharmacology, inspiring further interdisciplinary collaboration to solve the most pressing challenges in human health.

